# Mitochondrial Dysfunction and Multiple Sclerosis

**DOI:** 10.3390/biology8020037

**Published:** 2019-05-11

**Authors:** Isabella Peixoto de Barcelos, Regina M. Troxell, Jennifer S. Graves

**Affiliations:** Department of Neurosciences, University of California San Diego, San Diego, CA 92093-0935, USA; ibarcelos@ucsd.edu (I.P.d.B.); rtroxell@ucsd.edu (R.M.T.)

**Keywords:** multiple sclerosis, mitochondria, neuroinflammation, neurodegeneration

## Abstract

In recent years, several studies have examined the potential associations between mitochondrial dysfunction and neurodegenerative diseases such as multiple sclerosis (MS), Parkinson’s disease and Alzheimer’s disease. In MS, neurological disability results from inflammation, demyelination, and ultimately, axonal damage within the central nervous system. The sustained inflammatory phase of the disease leads to ion channel changes and chronic oxidative stress. Several independent investigations have demonstrated mitochondrial respiratory chain deficiency in MS, as well as abnormalities in mitochondrial transport. These processes create an energy imbalance and contribute to a parallel process of progressive neurodegeneration and irreversible disability. The potential roles of mitochondria in neurodegeneration are reviewed. An overview of mitochondrial diseases that may overlap with MS are also discussed, as well as possible therapeutic targets for the treatment of MS and other neurodegenerative conditions.

## 1. Introduction

Neurodegenerative diseases are characterized by neurologic dysfunction with a progressive course and consequent neuronal death [1]. Although these diseases, including multiple sclerosis, Alzheimer’s disease, and Parkinson’s disease, have different physiopathologies in their onset, they have a similar eventual course of gradual neurological decline and neuronal loss [2]. 

Multiple sclerosis (MS) is a leading cause of neurologic disability in young adults. MS is characterized by focal areas of demyelination in the white matter of the central nervous system (CNS) with secondary neuroaxonal degeneration [3,4]. The mean age of onset in females is approximately 30, compared to 33 years in males [5]. The sex ratio is 3:1, female to male, though men often progress more quickly and experience more rapid disability accumulation [3]. 

Among all patients with MS, about 85% present with a relapsing remitting form, which has alternate periods of acute demyelination (relapses) and periods of neurological recovery and stability (RRMS). For most patients, after 15–20 years the disease passes into a secondary progressive course (SPMS) which is characterized by an insidious progression of worsened neurological function with few or no acute relapses [3,4]. The remaining 10–15% of patients progress continuously from the first clinical manifestation of symptoms [4]; this is called the primary progressive form of multiple sclerosis (PPMS) and presents later in life, with a mean age of 45 years. The incidence of this form of the disease is approximately equivalent for men and women [6].

At the pathophysiological level, MS is characterized by two phases: At the initiation of a new lesion, there is a predominance of acute inflammation; subsequently, a state of chronic inflammation ensues with neurodegeneration. During the former, there is penetration of the blood brain barrier by activated immune cells against the myelin sheath. Inflammation in MS is due in part to components of both the innate and adaptive immune systems [7,8]. In brief, there is proliferation and dysregulation of pro-inflammatory T lymphocytes (Th1 and Th 17), as well as activation of B cells and secretion of inflammatory cytokines [9].

Pathognomonic inflammatory events in MS also activate neurodegenerative processes that lead to the destruction of oligodendrocytes, axons, and ultimately, neurons [7,8]. Brain, spinal cord and retinal atrophy are the result of the presence of neurodegeneration even at early stages of MS, meaning that both processes of acute inflammation and neurodegeneration co-exist since the first symptoms of the disease in the gray and white matter [3]. RRMS has a more prominent neuro-inflammatory phenotype, while the SPMS and PPMS forms are largely characterized by neurodegeneration [3]. 

The current available treatments for MS are directed against the acute episodes of neuroinflammation; this works well to prevent relapse events but is an approach with limited efficacy for protection against neurodegeneration, particularly in progressive forms of the disease [4]. The recent advancements in the understanding of mitochondrial dysfunction in neurodegenerative diseases, and in particular MS, bring new perspectives for future prevention of neuronal loss. We herein review the multifaceted role of mitochondria in MS pathology and the unique genetic factors that may contribute to the disease. 

## 2. Mitochondria and Their Role in Neurodegeneration in Multiple Sclerosis

### 2.1. Mitochondria

Mitochondria uniquely have dual genomic expression of proteins that originate from both nuclear and mitochondrial DNA (mtDNA) [10]. The multi-copy nature of mitochondria gives rise to the concept of heteroplasmy (when both mutated and wild-type mtDNA molecules coexist in the same cell) and homoplasmy (when only mutant mtDNA molecules are present in the mitochondria of the cell). For a disease to manifest symptoms, the mutated mtDNA molecules in a tissue must increase to a pivotal threshold beyond which oxidative phosphorylation (OXPHOS) is impaired, thereby demonstrating a critical ratio of mutant to wild-type mtDNA [11,12,13]. The mitochondrial genome (5-μm circles, or 16.569 kilobases) is smaller than the nuclear genome, is highly compacted, and has only one DNA polymerase (polymerase γ) without any introns [13,14]. The lack of protective histones facilitates the accumulation of mtDNA mutations in an environment with a high concentration of reactive oxygen species (ROS) [15]. 

Mitochondria are organelles which are responsible for cellular bioenergetics via the Krebs cycle (with the production of NADH and FADH) and oxidative phosphorylation (OXPHOS), for cellular bioenergetics with secondary ATP production [16,17]. The mitochondria’s main functions in bioenergetics include acting upon the electron transport chain (ETC) on the inner mitochondrial membrane, which is composed by four complexes (complex I, II, III and IV). They are also involved in the sequential reaction of reduction, OXPHOS, and electron flow (derived from NADH and FADH). This causes energy release which is used to transport protons from the matrix to the intermembrane space, creating an electrochemical gradient. ATP synthase (considered the complex V) uses this gradient to phosphorylate ADP to ATP [4]. 

Mitochondria participate in other crucial cells functions including calcium (Ca^2+^) storage, cell signaling (proliferation, adaptation to different environments and stress response) and apoptosis [18,19]. Ca^2+^ storage in mitochondria is involved in the regulation of ion homeostasis, cell signaling, and apoptosis (when prolonged high levels of Ca^2+^ in plasma concentrations) [20]. 

Also, important to understanding the potential role of mitochondria in neuronal death is the regulation of mitochondrial outer membrane permeabilization (MOMP). This is well controlled by different mechanisms, and when significant permeation does occur there is an activation of caspases and a release of pro-apoptotic factors into the cytosol, initiating the apoptosis-cascade [21,22,23,24,25,26].

### 2.2. Inflammation and Glia in Multiple Sclerosis

During acute events of inflammation, immune cells (mainly CD4+ T helper lymphocytes and also CD8+ T cells) cross the blood–brain barrier (BBB) and B cells and monocytes are activated. The primary target in MS is the myelin sheath of CNS white matter, though in recent years there has been growing evidence of direct attack against cortical and deep gray matter [27]. The release of pro-inflammatory cytokines (e.g., IL-17, IL-4, IL-10, TNF-α), activation of microglia and macrophages with the release of toxic substances such as reactive oxygen species (ROS), tumor necrosis factor, reactive nitrogen species (RNS), and glutamate [28] further damage the myelin. Enzymes involved in this neuroinflammation include myeloperoxidase, xanthine, NADPH oxidases (responsible for neuronal injury) [29,30], excitotoxins, cytotoxic cytokines, proteases and, lipases [31]. 

The damaged BBB subsequently becomes increasingly permeable, allowing further migration of immune cells leading to the formation of plaques of focal demyelination [32,33]. Focal plaques may converge, forming confluent demyelinated areas in both the white and grey matter [34]. The lymphocytic neuroinflammation process that characterizes the acute phase of the disease leads not only to the damage of myelin fibers synthesized by oligodendrocytes, but also to the death of the oligodendrocytes. The combination of demyelination and loss of trophic stimuli of oligodendrocytes then progresses to axonal degeneration, axon and neuron death with permanent neurologic disability [35]. Remyelination, if it occurs, is often only partial and astrocytes form sclerotic glial scars in the damaged white matter [36]. Chronic inflammation is also responsible for cumulative oxidation of phospholipids and DNA strand breaks [37]. 

In multiple sclerosis, there is also production of intrathecal, oligoclonal IgG and IgM. Although investigated extensively, no clear antigenic pattern identifying a specific potential trigger for MS has been found in studying these CSF antibodies. In SPMS there are also “meningeal lymphoid-like structures” that correlate with the pathology of the gray matter [38]. 

### 2.3. Neurodegeneration in Multiple Sclerosis and Evidence for Mitochondrial Involvement

Historically, the neurodegeneration of MS was understood as a sequential process following chronic neuroinflammation, but some evidence suggests that the neurodegenerative component is already present during the initial clinical manifestations of the disease [3]. The number of relapses in RRMS does not correlate with the probability or latency of progression of SPMS [39]. Tissue atrophy is considered an imaging marker of neurodegeneration in MS, and cerebral, spinal, and retinal atrophy have been reported to be present at the first clinical manifestations of RRMS, affecting both white and gray matter [40,41,42]. The accepted explanation for this observation is that neuroinflammation is followed by a failure in the process of remyelination, axonal damage and Wallerian degeneration [42]. Normal appearing white matter (NAWM), with normal macroscopic appearance and microscopically normal myelination, has a decreased density of axons; this is, in part justified by Wallerian degeneration, but also indicates more widespread early damage than captured by routine MRI [43,44,45,46,47,48]. Neuropathological findings from brain tissue blocks of MS patients show evidence of gray matter lesions (axonal and dendritic transection, apoptotic neurons and demyelinated cortical plaques) [49] present from the time of initial disease onset; this is, particularly prominent in SPMS and PPMS. 

The chronic neuro-inflammatory stimuli of MS disrupt neuro-axonal hemostasis, leading to a simultaneous increase in oxidative stress, marked by a rise in ROS, and secondary damage to mitochondria and macromolecules (mtDNA, proteins from ETC, lipids). Excitotoxicity and an imbalance of neurotrophic substances for neurons and oligodendrocytes occurs [3,50,51]. This damage impairs mitochondrial function (below described), which further increases ROS production in a vicious cycle [4]. The result is a reduction in the efficiency of energy production, creating an imbalance between energy generation and consumption. The final result is an environment with a failure to provide required levels of energy within the demyelinated axons, and after reduced ATP production reaches a critical point there is an imbalance in ionic homeostasis leading to activation of apoptosis mechanisms [52,53]. 

Of relevance to understanding the pathology of MS, the central nervous system (CNS) has increased susceptibility to oxidative damage because of the high metabolic rate (consumption of oxygen) of neurons and the rich composition of polyunsaturated fatty acids in CNS cells [54]. Furthermore, mitochondria influence the differentiation of oligodendroglial cells through overexpression of mitochondrial transcripts and mtDNA [55]. An environment of oxidative stress reduces the expression of these transcripts involved with oligodendrocyte differentiation [56]. Double strand breaks in mtDNA have been shown to cause an oligodendropathy and exaggerated injury responses in an animal model of MS [57]. Additional observations have demonstrated a potential direct link between mitochondrial dysfunction and oligodendrocyte myelination. N-acetyl aspartate (NAA), is a mitochondrial metabolite and also an indirect oligodendrocyte substrate for the production of myelin (after breakdown into acetate and aspartate). A lack in the availability of NAA, from damaged mitochondria, was associated with lower levels of acetate in the cortex (parietal and motor) in postmortem tissue from patients with MS [58]. 

Though they are often overridden and unable to counter the stress burden, there are compensatory mechanisms to counter these mitochondria-related degenerative processes. The body has intrinsic mechanisms of self-protection against ROS, including nuclear factor erythroid 2­related factor 2 (NRF2) and antioxidant enzymes such as heme oxygenase 1 (HMOX1) which are activated during periods of hypoxic stress [59]. But after a critical point in the reduction in ATP production, an imbalance in ionic homeostasis occurs, leading to the activation of apoptosis mechanisms mediated by ions (Ca^2+^ dependent proteases) in those axons with chronic inflammation and demyelination [60]. 

#### 2.3.1. Human Studies of Mitochondria Function in Multiple Sclerosis

**Figure 1:**
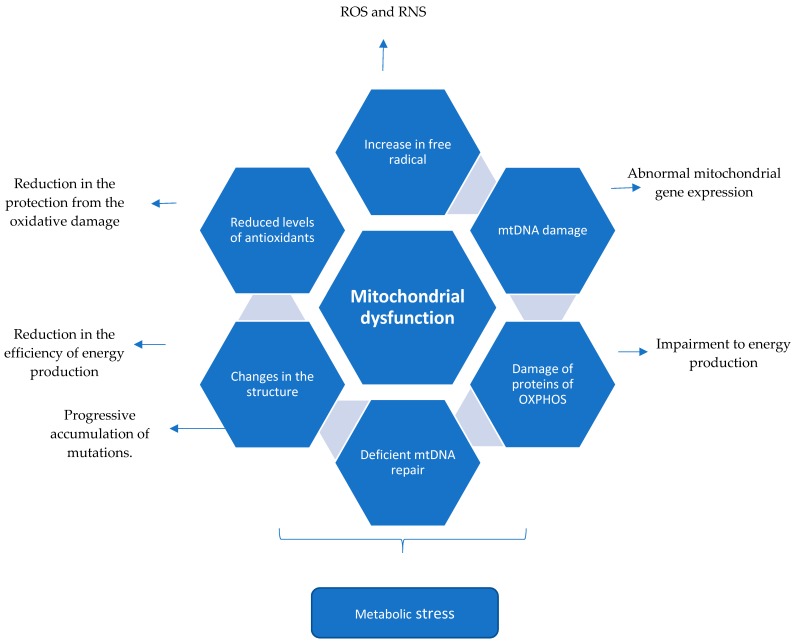
Mitochondrial dysfunction described in the literature associated with Multiple Sclerosis. Chronic neuroinflammation leading to mitochondrial dysfunction.

Multiple human studies have demonstrated evidence of mitochondrial dysfunction in MS patients (Figure 1, Table 1). One publication compared 10 post mortem brains of patients with MS (n = 9 SPMS and n = 1 PPMS) to healthy controls paired for age and sex. The MS cortex exhibited distinctive levels of both mtDNA transcripts (488 decreased and 67 increased compared to controls), and nuclear mitochondrial DNA transcripts (26 decreased transcripts). In a study of function in the same samples there was a decrease in complex I and III activity from the neurons of motor cortex in MS patients, and a decrease in GABAergic synaptic components [52]. Another study of thirteen patients with MS (SPMS) identified large mtDNA deletions in neurons, with some showing specific deletions in the subunits of complex IV [59]. 

Additionally, reports of a decrease in PGC-1α levels (a transcriptional co-activator and regulator of mitochondrial function) in pyramidal neurons of MS patients (7 SPMS, 7 PPMS and 1 subtype not determined) was associated with reduced expression of mitochondrial machinery components (OXPHOS subunits, antioxidants and uncoupling proteins 4 and 5). This finding was confirmed in a functional model (with neuronal cells) showing association of these changes with more ROS production [60]. Another publication reported that increases in ROS affect the ability of NRF-2 (a transcription factor for ETC proteins) to bind promotors, even in apparently normal areas of gray matter cortex of SPMS patients [61]. Higher ROS production in the CNS of progressive MS patients (14 SPMS, 5 PPMS and 7 subtypes not determined) has also been associated with a rise in the number of mitochondria in axon and astrotcytes. Increased ROS is also associated with the translation of mitochondrial proteins in active and chronic inactive MS lesions, including elevated expression of proteins from the mitochondrial ETC complex IV and higher levels of a heat shock protein (mtHSP70) compared to the brain of controls. The mtHSP70 protein is a marker of mitochondrial stress [62]. A recent publication found in fronto-parietal areas decreased levels of the potent antioxidant, glutathione (GSH), in PPMS and SPMS compared with RRMS and controls, suggesting that oxidative stress affects the neurodegeneration phase more than the neuroinflammatory phase [63]. Another study compared the mitochondrial proteome from the brains of MS (eight SPMS) patients and controls. The findings showed different patterns by mass spectrometry in levels of human cytochrome c oxidase subunit 5b (COX5b), the brain-specific creatine kinase isoform, and the β-chain of hemoglobin between groups [64]. 

#### 2.3.2. Neurodegeneration in Multiple Sclerosis Animal Models

There have been many attempts to reproduce the spectrum of inflammation (acute and chronic), demyelination/remyelination, and neurodegeneration that characterize the different clinical syndromes (PP, SP, RR) of the disease. There is no single experimental model that fully covers the spectrum of pathology in human MS. Each model available has strengths for certain questions, but without completely recapitulating all of the mitochondrial deficiencies in MS [67]. In trial design it is important to focus on the mechanism of the potential drug and choose the animal model in which it is possible to induce the disease process of interest [67,68].

In one of the most commonly used models for MS, experimental autoimmune encephalomyelitis (EAE), there are morphology changes in the mitochondria (swelling) [69], early mitochondrial dysfunction even in normal appearing white matter [70], and impairment of mitochondrial and axonal depolarization [71]. Some of the mitochondrial damage can be rescued with specific interventions such as gene therapy for expressing complex I ETC proteins [72,73] and antioxidant cocktails [74,75]. In a myelin basic protein (MBP) knockout, considered a model for the chronic demyelination of MS, there were increased numbers of mitochondria observed by electron microscopy. Additionally, there was a two-fold increase in the cytochrome c staining in the white matter, showing mitochondrial changes associated with cases of reduction in myelin [76]. A summary of previous animal models’ findings regarding the association of mitochondrial involvement in multiple sclerosis is presented in Table 2. 

### 2.4. Summary (Mechanism of Mitochondrial Dysfunction Perpetuating the CNS Injury in Multiple Sclerosis) 

**Figure 2:**
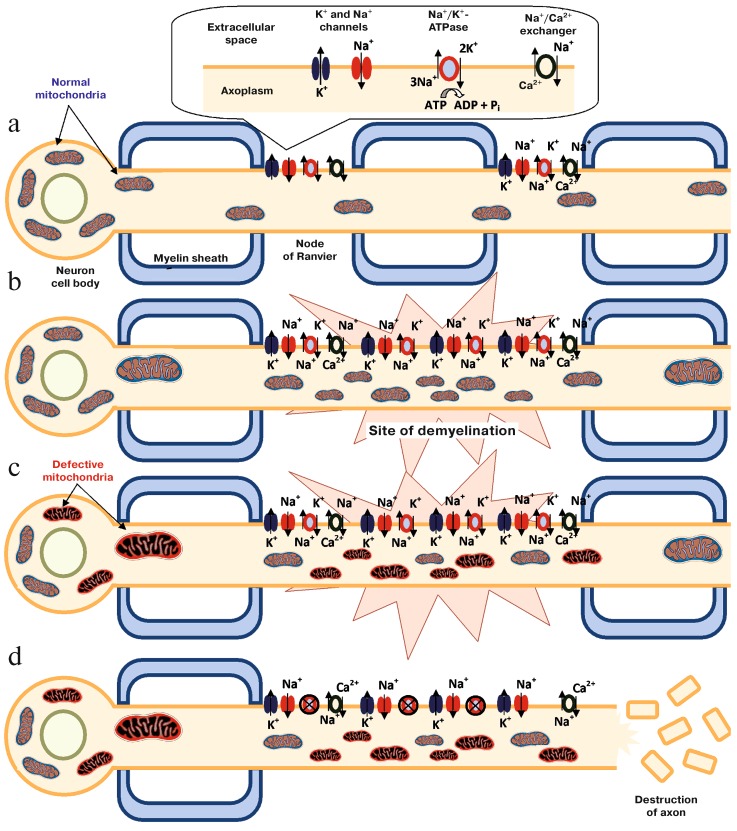
The role of mitochondria in the process of neurodegeneration. **a**. Normal nerve. **b**. Site of demyelination with secondary modification of the distribution of ion channels in the nerve. **c.** Structural and functional modification in mitochondria caused by oxidative stress. **d**. Cascade of apoptosis activated by Ca^2+^. Figure reprinted with permission from the article “Involvement of Mitochondria in Neurodegeneration in Multiple Sclerosis”, Kozin et al., Biochemistry (Moscow), 2018, Vol. 83, No. 7, pp. 813–830 [4].

The brain has a high metabolic rate and consumes 20% of the total energy produced in the human body, which is mainly utilized in neurotransmission (more than half of that consumed to maintain the ionic equilibrium and the membrane potential) and the axoplasmic flow (to conduct nerve impulses); these functions depend substantially on mitochondria machinery [4,85]. The neurologic signal transmission is due to propagation of the membrane depolarization through the neuron, and the electrochemical gradient is created by the Na^+^/K^+^-ATPase, allocated in the nodes of Ranvier. Oligodendrocytes are not only responsible for the myelin sheath but also release lactate for the neuron as energy supply. With the chronic inflammation and myelin destruction, there is redistribution of the ion channels. Consequently, there is more ATP consumption by the increased number of Na^+^/K^+^-ATPase. With the purpose of balancing the ratio and demand for energy, mitochondria begin compensatory modifications (increasing in number and size, changing the localization in the neuron and its morphology). In parallel, the chronic inflammation creates an environment of oxidative stress secondary to ROS release by macrophages and the microglia and increases in glutamate released in response to neuronal damage. TNF-α damages the OXPHOS process through Ca++ regulated mechanisms [86]. With mitochondrial progressive accumulative damage (mtDNA alteration and increased heteroplasmy, OXPHOSP subunits dysfunction, alteration in proteins that regulates the migration of the organelle from neuron body to the axon) significant impairment in energy production develops. [51]. If ATP production is compromised, the Na^+^/K^+^-ATPase is not able to keep the gradient after an action potential, which leads to Na+ accumulation in the neuron cytoplasm. This forces the Na^+^/Ca^2+^ channel to transfer Ca^2+^ inside the cell, activating the Ca^2+^ apoptosis-depend-cascade, which results in neuron death, Wallerian degeneration and irreversible neurologic dysfunction [34,53,65,66,86,87,88,89]. This process is represented in Figure 2. The progressive degenerative process initiated in the axon can continue to the neuron body and dendrites, also reaching presynaptic and postsynaptic neurons [34], chronic failure to provide energy to the tissue increases the oxidative stress in a vicious cycle that increases mitochondrial damage [51]. 

Important to mention is that mitochondrial DNA damage is amplified during the process of expansion of the clones (with deletions or mutations), changing the levels of heteroplasmy of the tissue [51,59]. This process increases the failure to provide appropriate energy supply for the tissue, contributing to the death of the cells [90].

## 3. Mitochondrial Mutations in Multiple Sclerosis and Overlapping Diseases

### 3.1. Mitochondrial Mutations and Multiple Sclerosis Risk

The current consensus is that MS is a multifactorial disease, with 25% of the risk related to heritable factors [91]. The important role of the class II region of the human leukocyte antigen (*HLA*) gene cluster has been well recognized for several decades. There are now over 100 loci identified in the HLA region found to be associated with susceptibility and over 200 in non-HLA loci [92]. Several single site mutations in mtDNA have been reported to increase the risk of MS, including the mtDNA nt13708A [93] and mtDNA T4216C [94] variants. A large consortium study by Tranah et al. examined mitochondrial DNA sequence variation and MS risk. In the discovery dataset they compared over 7000 MS cases and over 14,000 controls from seven countries. Haplotype group and more than 100 common mtDNA mutations were evaluated. While they reported an elevated risk of MS (OR 1.15, *p* = 0002) among haplotype JT carriers, they found no associations between common mtDNA mutations and MS risk [95].

### 3.2. Leber’s Hereditary Optic Neuropathy

Leber’s hereditary optic neuropathy (LHON) is a mitochondrial disease resulting in severe bilateral optic neuropathy, characterized by central vision loss and dyschromatopsia. There is degeneration of the retinal ganglion cells (RGC) and axonal tracts of the optic nerve. There are numerous mitochondrial mutations associated with LHON, but the vast majority of patients have one of three different mitochondrial mutations at nucleotides 3460, 11778 and 14484. The mutations are single amino acid substitutions in one of the mitochondrially encoded subunits of NADH: ubiquinone oxidoreductase, complex I of the electron transport chain (ETC). There is some evidence suggesting that the exposure to high nitric oxide concentrations could impair in vivo the ability to cope with the oxidative stress caused by the genetic defect, thereby driving the pathology in LHON. This was described by Flabella et al. in one patient carrying the 11788/ND4 mutation [96]. This same increase in ROS was described in MS as previously discussed [3,50,51].

Males are more frequently affected, and there is incomplete penetrance seen in LHON families [97]. There is a modest epidemiological overlap between MS and LHON, with a subset of patients developing both diseases (Table 3). Harding first described this association in 1992 in case studies of eight women with matrilineal relatives with LHON who presented with optic neuritis; six of the eight progressed to clinical MS with neurologic symptoms. Seven of the eight also had characteristic white matter lesions on MRI [98]. Since Harding’s first report of the association of LHON and MS this relationship has continued to be observed with females being predominantly affected at a ratio of more than two to one [99]. In one review the incidence of demyelination among LHON affected persons was up to five percent, which is fifty times greater than the prevalence of MS in the general population [100]. RGC thinning is also noted in MS. While the exact pathophysiology may be different in LHON and MS, the mitochondrial dysfunction in LHON may be instructive to the understanding of mitochondria’s role in MS.

### 3.3. Dominant Optic Atrophy and OPA1 Mutations

An additional example of potential overlap between mitochondrial genetic optic atrophy and MS has been described by Yu-Wai-Man et al. in a paper detailing three cases of MS-like disease associated with *OPA1* mutations (Table 3). *OPA1* mutations have previously been discussed in the literature in association with autosomal dominant optic atrophy (DOA), the most common inherited form of optic nerve visual loss. OPA1 has multiple roles in mitochondrial function as it encodes for an inner mitochondrial membrane protein, and is involved in respiratory chain complexes, cytochrome c molecules, and fusion/fission balance. There are over 90 known gene mutations (substitutions, deletions and insertions) associated with OPA1 mutations and thought to be due to a truncated protein [101]. Like LHON, DOA is a mitochondrial determined optic neuropathy preferentially affecting the ganglion cells within the inner retina. The exact relationship between *OPA1* proteins and MS has yet to be clearly elucidated, with only the above few cases being reported.

### 3.4. POLG1 Mutations

The mitochondrial gene *POLG1* is the larger catalytic subunit of polymerase gamma which is the only known DNA polymerase active in human mitochondria. *POLG1* mutations have been implicated in a number of mitochondrial disorders and more recently have also been identified in several cases of demyelination. Two cases of non-related individuals with novel *POLG1* mutations who had optic neuritis and white matter lesions consistent with clinical MS were reported in the literature. Of note both of these patients progressed into a more classic POLG1 phenotype with bilateral ophthalmoplegia, ptosis, myopathy, cardiomyopathy, ataxia, dysphagia, and hearing and cognitive impairment. These patients also had muscle biopsies showing red ragged fibers [99,102]. Therefore, their progression calls into question whether or not they truly had MS or if their initial presentations were instead MS mimics. Clearly more research is needed. Yet it is important to consider these cases as they may offer further evidence of the role of the mitochondria within MS and MS-like disease processes. Further research may lead to the discovery of more MS patients with mitochondrial mutations.

## 4. Potential Therapies and Targets

The treatment of most mitochondrial diseases is still largely supportive at this time, although some therapies have been tried such as vitamins, co-enzymes, creatine, free radical scavengers and hyperbaric oxygen treatments. Despite the widespread use of a multitude of co-enzymes and vitamin supplements there is currently limited evidence that these are effective in the treatment of primary mitochondrial disorders. For targeted treatment of MS the use of alpha lipoic acid and co-enzyme Q10 are being investigated. A randomized controlled phase 2 trial of alpha lipoic acid (ALA) versus placebo was studied in SPMS and found to slow whole brain atrophy [103]; further studies are ongoing. Other studies are examining a synthetic analogue of co-enzyme Q10, idebenone, which is being targeted for treatment of neurodegenerative disorders such as LHON [99]. Phase I/II trials of idebonone in PPMS demonstrated safety but initial data showed no change in progression of the disease (http://www.santhera.com/assets/files/press-releases/2018-03-05_PR_PPMS_e_final.pdf). An expansion study is ongoing with completion planned for later this year (https://www.clinicaltrials.gov/ct2/show/NCT01854359). In addition to these targets there are several other potential approaches for mitochondrial based therapy and limiting neurodegeneration in MS.

### 4.1. Mitochondrial Metabolism and Chronic Neuroinflammation

Neurodegeneration is in part driven by the activation of mononuclear phagocytes. When mononuclear phagocytes are persistently activated it can lead to a state of chronic neuroinflammation. Mitochondrial metabolism has a role in the inflammation cascade and targeting the metabolism of innate immune cells may be of benefit. Future studies may address this relationship to aid in the development of novel molecular and cellular therapies that could disrupt the state of chronic neuroinflammation as a way of preventing secondary neurologic damage [104]. Therapies that support cellular metabolism such as high dose biotin, iron and vitamin D have been proposed as possible treatment therapies in progressive MS, and studies looking at each of these treatments are ongoing (https://clinicaltrials.gov). The pilot studies of high dose biotin are encouraging and results suggest both a reduction in disease progression as well as decreased disability in PPMS [105]. Furthermore, these therapies may also have a role in preventing progression of RRMS to SPMS [50].

### 4.2. Gene Therapy

Gene therapies are being developed in mitochondrial disorders, though most are still in early phases of development. In vivo studies in mice using several vectors have been promising in some disease models such as LHON [106]. Gene Therapy GS010 was shown to be safe in LHON patients carrying the G11778A mutation in a phase 1/2 clinical trial (https://clinicaltrials.gov/ct2/show/NCT02064569). Although the results did not have sufficient power to definitively demonstrate efficacy, 6/14 patients who received GS010 had visual acuity improvements [107]. While these early results in LHON do not immediately translate to MS care, the suggestion of treatment effect is promising for the future of gene therapy in this field of mitochondrial dysfunction.

## 5. Conclusions

There is compelling data to suggest an important role for mitochondria in the pathophysiology of MS. Further work is needed to move from studies of association to understanding causal relationships between failure of mitochondrial function and MS phenotype. Targeting energy failure and mitochondrial dysfunction is a novel potential therapeutic approach for the challenging progressive phase of MS. Trials are already underway to begin exploring these pathways as treatment targets, including studies of biotin and alpha lipoic acid in progressive MS.

## Figures and Tables

**Table 1:** Evidence of mitochondrial involvement in Progressive Forms of Multiple Sclerosis compared to Controls or RRMS.

MS Phenotype	Tissue	Cell Type	Mitochondria Pathology	Reference
1 PP 9 SP 8 C	Motor cortex	Neurons	—decreased expression of mitochondrial nuclear gene DNA —functionally reduced complex I and III activities	Dutta, R. et al. 2006 [52]
1 PP 9 SP 6 C	Chronic inactive lesions	Demyelinated axons	—increased total mitochondrial content and complex IV activity	Mahad, D.J. et al. 2009 [65]
8 SP 5 C	Grey matter in Cortex	NCD	—epigenetic changes affected by ROS, through the reduced capacity of NRF-2 (a transcription factor for ETC proteins)	Pandit, A. et al. 2009 [61]
5 PP 14 SP 7 ND 7 C	Active and chronic lesions	NCD	—increase in the levels of a heat shock protein (mtHSP70), a marker of mitochondrial stress —an increase in the number of mitochondria and in the translation of mitochondrial proteins	Witte, M.E. et al. 2009 [62]
13 SP 10 C	NCD	Neurons	—accumulation of large mtDNA deletions, with some showing specific deletion in the subunits of complex IV	Campbell, G.R. et al. 2011 [59]
2 PP 7 SP 1 RC	NCD	Acute and chronic demyelinated axons	—increased mitochondrial content and complex IV activity compared with remyelinating and myelinated axons	Zambonin, J.L. et al. 2011 [66]
8 SP 8 C	NCD	NCD	—different patterns of mass spectrometry in human cytochrome c oxidase subunit 5b (COX5b), the brain-specific creatine kinase isoform, and the β-chain of hemoglobin	Broadwater, L. et al. 2011 [64]
7 PP 7 SP 1 ND 9 C	NCD	Pyramidal neurons	—decrease in PGC-1α levels, OXPHOS subunits, antioxidants and uncoupling proteins 4 and 5	Witte, M.E. et al. 2013 [60]
20 PP 20 SP vs 21 RR	NCD	NCD	—decreased levels of glutathione (GSH), a potent antioxidant, signaling that oxidative stress more strongly affects the neurodegeneration phase than the neuroinflammation one	Choi, Y. et al. 2018 [63]

MS Type: PP = primary progressive; SP = secondary progressive; RR = relapsing progressive; C = controls; ND = not determined; NCD = Tissue or Cell Type not clearly defined.

**Table 2:** Mouse models to study multiple sclerosis [77].

MS Animal Model	Type of MS Modeled	Indication for Research	Mitochondrial Findings
EAE-SJL/J mice -C57BL/6J mice -Biozzi chronic EAE	-RR -PP and SP -RR -> SP	Understanding of the neuroinflammatory process after immunologic activation of the mice (SJL/J with PLP or MBP and C57BL/6J with MOG) [77,78]. Accumulative damage of neuroinflammation with secondary progression of the disease [68,79,80].	C57BL/6′s mitochondria morphology changes (swelling) [69], early mitochondrial dysfunction in EAE disease [70] and impairment of mitochondrial and axonal depolarization [71]. C57Bl/6 model did not reproduce the cortex respiratory protein’s alterations seen in MS patients [64].
TCR transgenic mice	-RR [78]	Understanding spontaneous neuroinflammatory process after immunologic activation [77].	-
TMEV	Demyelination and axonal damage	Infection mediated by Picornavirus inducing an encephalomyelitis (whole neuroaxis) [77].	-
Toxin-induced demyelination (Cuprizone, Lysolecithin, Ethidium bromide)	Demyelination and remyelination	Reproducible onset of demyelination and start of remyelination after interruption of toxic exposure. If chronic exposure of cuprizone also possible to see impairment of remyelination [81].	Cuprizone is a copper chelator an essential component of COX [82]. Mice’s brain treated with cuprizone presented “giant” mitochondria in oligodendroglial cells [83]. Oligodendrocytes treated with cuprizone presented with decreased mitochondrial potential (in vitro) [84].

EAE= experimental autoimmune/allergic encephalomyelitis. TCR transgenic mice = T cell receptor (TCR) transgenic mouse models. TMEV = Theiler’s murine encephalomyelitis virus. PLP = proteolipoprotein. MBP = myelin basic protein. MOG = myelin oligodendrocyte glycoprotein. COX = cytochrome oxidase.

**Table 3:** Associations between MS and mitochondrial diseases.

Disease	Gene Mutation	MS Overlap	Overlap in Potential Mechanism
**MS**	mtDNA nt13708A mtDNA T4216C nt 11778 (G→A)	NA	NA
**LHON**	nt 3460 nt 11778 (G→A) nt 14484	5% LHON have evidence of demyelinating lesion	Degeneration of optic nerve
**DOA**	over 90 gene mutations	*OPA1* protein: known link to DOA, implicated in 3 patients with MS-like disease	*OPA1* mutation and truncated protein
***POLG1***	Not specified	Linked to cases of demyelination	Not specified

MS: multiple Sclerosis, LHON: Leber’s hereditary optic neuropathy, DOA: autosomal dominant optic atrophy, *POLG1:* mitochondrial gene *POLG1.*
